# Malarial Fevers Treated by Quiniæ Sulph

**Published:** 1886-07-20

**Authors:** John H. Young

**Affiliations:** Roswell, N. M.


### MALARIAL FEVERS TREATED BY QUINIÆ SULPH. INUNCTIONS.

By JOHN H. YOUNG, M. D., ROSWELL, N. M.

There are times when we stand by the bed of our patients totally cast down by the knowledge that the introduction into the system of quiniæ sulph. alone holds out a shadow of hope. The stomach is irritable, and will retain it in no combination. Rectal injections have been faithfully tried and failed, and our last resort is gone. The patient is doomed ; the friends and relatives look to you, a "broken reed," for help in their hour of trouble, and then it is that our life-work becomes intolerable and we regret the day that decided us to be disciples of Æsculapius !

This has been, in the above named disease, the misfortune of myself and, doubtless, of others. With me, the day has passed ; no more do I fear the falling of that misfortune.

In the early months of my practice, I frequently, in treating children, owing to the difficulty of getting them to take the remedy uncombined, ordered quiniæ sulph. rubbed up with lard and well rubbed into the groins, axilla, and over the abdomen. The result was so satisfactory that, from using it as a tonic, I tried it in malarial troubles with children, and then with adults. Now, after two year's use of it in that way, I unhesitatingly say that I have just as much confidence in quiniæ sulph., applied by inunction, as when given per orem or per rectum.

I do not offer this *method* as a specific remedy in these or any other troubles; but in my hands it has been of the greatest assistance—not that the effect of the drug is intensified, but that it gives you the full systemic effect without the slightest derangement of the stomach or bowels—a result so frequently resulting from its long continued use in full doses. It also gives you the minimum cerebral derangement.

In order to give you clearly an idea of the action of the remedy thus applied, I cite the case of Mrs. Y——, just dismissed. Others could be added, but one will suffice:

May 27, a.m., temperature 101°; pulse 92; tongue furred and dry; urine scanty and highly colored; bowels constipated. ℞. Hydrg. chl. mit., grs. iij; P. sacch. alb., grs. x. M. Ft. Pulv. No. iij. Sig. One every hour. ℞. Pot. acct., ʒ ij; aquæ, fl. ℥ iij. M. Sig. ℥ ss. every four hours; also ordered two 10-grain capsules of quiniæ sulph. to be given in the morning at intervals of two hours.

May 28, a. m. Temperature 103°; pulse 96; tongue and urine unchanged; stomach very irritable, and unable to retain quiniæ; bowels tympanitic, and inclined to diarrhœa. ℞. Hydrg., chl. mit., P. opii, aa grs. iv. M. ft. pulv. No. iv. Sig. One every three hours. Ordered the abdomen rubbed with a liniment of turpentine, vinegar and egg, and ℈ j of quiniæ sulph. per rectum at 9 o'clock in the morning.

May 29, a. m. Temperature 101°; pulse 92; tongue slightly furred; urine unchanged; bowels moving every hour; tympanitis unchanged. ℞. P. opii, grs. iv; bismuthi sub nit. ℈ ij. M. ft. pulv. No. iv. Sig. One every two hours. Local application to bowels continued. Evening—Temperature 104¼°; pulse 98; tongue and urine unchanged; diarrhœa checked. Ordered quiniæ sulph. ʒ ss, tr. opii, gtts. xx, per rectum for next morning.

May 30, a. m. Temperature 102½°; pulse 90; otherwise no change. Evening—Temperature 104¾°; pulse 99; tymphanitis increased, with soreness over abdomen, and vomiting; bowels again loose. Ordered opium for diarrhœa, and spiced poultice to bowels. For morning: ℞. Quiniæ sulph. ℈ iijss; adepis q. s. M. Sig. Rub well on lower abdomen and thighs.

May 31, a. m. Temperature 102°; pulse 92; bowels and stomach better. Poultice continued. Evening—Temperature 102°; diarrhœa controlled; tenderness of abdomen and tympanitis disappearing; tongue clearing. Inunction of quiniæ to be repeated in the morning.

June 1, a. m. Temperature 99¾; pulse 88; tympanitis nearly gone, and tenderness entirely disappeared. Evening—Temperature 100; pulse 90 ; other symptoms unchanged. ʒ i quiniæ sulph. for morning inunction, and lemonade to be drunk freely.

June 2, a. m. Temperature normal; pulse 88; patient pronounces herself much better. Evening—Temperature 99°; pulse 88. Quiniæ sulph. ℈ ij by inunction next morning.

June 3, p. m. Temperature normal.

June 4. No rise since yesterday morning, and patient desirous of sitting up.

In conclusion, I will say that I have given in two doses, at interval of two hours, ℈ v of quiniæ by inunction without, in the least, affecting the stomach, and with no more cerebral disturbance than I have seen result from ℈ ss dose per ovem.

